# Combining Transcriptomics and Polyphenol Profiling to Provide Insights into Phenolics Transformation of the Fermented Chinese Jujube

**DOI:** 10.3390/foods11172546

**Published:** 2022-08-23

**Authors:** Cheng Wang, Peiyao Li, Beibei Zhang, Xiang Yu, Xingang Li, Gang Han, Yamei Ren, Jingfang Zhang

**Affiliations:** 1 College of Forestry, Northwest A&F University, Xianyang 712100, China; 2 College of Chemistry and Chemical Engineering, Xi’an University of Science and Technology, Xi’an 710054, China; 3 Center for Jujube Engineering and Technology of State Forestry Administration, Northwest A&F University, Xianyang 712100, China; 4 College of Food Science and Engineering, Northwest A&F University, Xianyang 712100, China

**Keywords:** Chinese jujube, *Monascus purpureus*, polyphenol compounds, carbohydrate-active enzymes, liquid state fermentation

## Abstract

As an important medicine homologous food, Chinese jujube is rich in nutrition and medicinal value. To enhance the bioactive compounds level of Chinese jujube products, three kinds of fungi strains (*Rhizopus oryzae*, *Aspergillus niger* and *Monascus purpureus*) were firstly selected to evaluate their effects on total soluble phenolic compounds (TSPC) and total soluble flavonoids compounds (TSFC) contents during liquid state fermentation of Chinese jujube. As the best strain, the highest contents of TSPC and TSFC could increase by 102.1% (26.02 mg GAE/g DW) and 722.8% (18.76 mg RE/g DW) under *M. purpureus* fermentation when compared to the unfermented sample, respectively. Qualitative and quantitative analysis of individual polyphenol compounds indicated that proto-catechuic acid, *p*-hydroxybenzoic acid and chlorogenic acid showed the highest level in the fer-mented Chinese jujube at the 7th day, which was enhanced by 16.72-, 14.05- and 6.03-fold when compared to the control, respectively. Combining with RNA sequencing, function annotation of CAZymes database and polyphenol profiling, three potential transformation pathways of poly-phenol compounds were proposed in the fermented Chinese jujube by *M. purpureus*, such as the conversion of insoluble bound phenolic acids, rutin and anthocyanin degradation. These findings would be beneficial for better understanding of the biotransformation mechanism of polyphenol compounds in fungi fermentation.

## 1. Introduction

Chinese jujube (*Zizyphus jujuba* Mill.), a fruit of the dicotyledonous Rhamnaceae plant, is widely distributed in the subtropical and tropical regions of Asia and America, as well as the Mediterranean [1]. According to the previous reports, Chinese jujube contains a variety of bioactive components, such as polysaccharides, phenolics, triterpenoids and cyclic nucleotides [2,3]. The recent pharmacological studies have confirmed that Chinese jujube possesses various bioactive activities, including anticancer, antioxidant activities, anti-epileptic, anti-insomnia, and so on [4]. As the only country exporting jujube, notably, China could not only own the most variety of jujube cultivars around the world, but also accounts for 90% of the world’s jujube production [5]. The annual production of fresh Chinese jujube had increased continuously to over 15 million tons due to the demand for food and pharmaceutical applications as early as 2018 [6]. Therefore, how to explore the functional products with higher bioactivities would be very important for the development of the Chinese jujube industry.

In the previous studies, it had reported that Chinese jujube possessed the highest phenolic content and antioxidant activities among 62 common fruits [7]. As an important bioactive compound in Chinese jujube, polyphenol compounds existed with different types: free, soluble conjugate and insoluble bound forms [8]. Thus, insoluble bound polyphenol compounds (ISPC) are covalently conjugated to cell wall components, such as polysaccharides, cellulose, pectin and lignin [9]. However, the lower utilization of ISPC has seriously affected the exploitation and application of these bioactive compounds in food materials. Therefore, some technologies have been developed to solve this problem. Thus, many researchers have verified that microbial fermentation could improve the liberation of ISPC effectively by generating some carbohydrate-hydrolyzing enzymes (e.g., *α*-amylase, cellulase, *β*-glucosidase and xylanase) [9], and further improving nutritional qualities or biological activities of food, agro-industrial residues or plants [10,11,12]. For example, it has been reported that *Aspergillus niger* fermentation has exhibited great potential in increasing the bioactive compounds of food or tartary buckwheat leaves [13]. The extracts of the fermented rice bran by *Lactobacillus rhamnosus* and *Saccharomyces cerevisiae* showed strong melanogenesis inhibition activity [14]. Fermentation with yeast or lactic acid bacteria was helpful to improve the contents of the extractable phenolic compounds, folates and free ferulic acid in rye [15].

As highly efficient producers of many extracellular enzymes (e.g., cellulases, pectinases and amylases), fungi (e.g., *Monascus*, *A. niger*, *Rhizopus oryzae* and *Rhizopus oligosporus*) have been widely applied in food or agro-industrial residues fermentation and large-scale production of bio-based chemicals (e.g., organic acids, pigment and bioactive compounds) [13,16,17,18]. Currently, many studies of fungi fermentation mainly focus on the action of solid-state fermentation (SSF). They mainly investigate the effects of different fungi strains on the release of bioactive compounds (e.g., polyphenol) and bioactivities enhancement in some food materials, including black rice bran (*Aspergillus awamori* and *Aspergillus oryzae*) [14], oats (*Monascus anka*) [10], guava leaves (*Monascus anka* and *Bacillus* sp.) [19] and so on. However, the limitation of moisture is a key influencing factor to allow the microbial growth and metabolism in SSF (especially for filamentous fungi), which was very difficult to control in large-scale fermentation. Compared with SSF, liquid-state fermentation (LSF) has many advantages, such as the controllability of fermentation parameters, higher productivity and shorter fermentation time [20]. Meanwhile, it has been confirmed that LSF is the main method for enzyme production (e.g., cellulase) because of easy parameter control and a good technological basis for scaling to industrial level [21]. Therefore, LSF is widely applied in processing of some food materials to produce various bio-functional products, such as kiwifruit [22], noni fruits [23], grape waste [12] and so on. Nevertheless, little is known about the potential transformation or degradation mechanism of bioactive compounds (e.g., polyphenols) during the processing of LSF.

In this work, three kinds of fungi strains were selected to estimate their effects on the biotransformation of polyphenol compounds in the liquid-state fermentation of Chinese jujube. Then, the fermentation conditions of the best strain were further optimized, and we investigated the changes of the composition and contents of phenolic compounds during the fermentation. Further, the potential conversion (or degradation) mechanism of polyphenol compounds was clarified based on the expression of CAZymes, enzymes activities determination and the changes of phenolic compounds. These results would provide useful information on the utilization of fungal fermentation to improve the bio-functional activity of Chinese jujube products.

## 2. Material and Methods

### 2.1. Materials, Reagents and Enzymes

Chinese jujube (the name of the cultivar is Hetian yuzao) was purchased from the local farmers’ market of Yangling (Shaanxi, China). The cultivar of Chinese jujube was first confirmed by the information on product trademark (including cultivar (Hetian yuzao), producing areas (Hetian county, Hetian City, Xinjiang province) and producing time (20 November 2021)), which was further validated by Professor Xingang Li in Northwest A&F University. The moisture contents of the samples were 17.3%. All of the samples were sealed and stored at −80 °C before use.

Gallic acid, quercetin, *p*-coumaric acid, ferulic acid, kaempferol and Folin-Ciocalteu phenol reagent were all purchased from Sigma-Aldrich (St. Louis, MO, USA). The rest of the standards of phenolic compounds (i.e., catechins, chlorogenic acid, rutin, protocatechuic acid, *p*-hydroxybenzoic acid, quercetin-3-*O*-glucuronide, hyperoside and luteolin) used in this work were all obtained from Solarbio (Beijing) Technology Co., Ltd. (China), Yuanye (Shanghai) Biotechnology Co., Ltd. (China) and Mobei (Shanghai) Biotechnology Co., Ltd. (China), respectively. All other chemicals and regents were analytical grade or HPLC grade and purchased in China.

### 2.2. Liquid-State Fermentation of Chinese Jujube

The fungi *Rhizopus oryzae*, *Aspergillus niger* and *Monascus purpureus*, all stocked in our laboratory, were used in this work. Three kinds of strains were all incubated on potato dextrose agar (PDA) medium for 7 days at 30 °C. The same seed and fermentation cultures were used for each strain. In brief, 5 mL of the spore suspension (approximately 10^7^ spores/mL) washed from the PDA medium with 0.9% NaCl solution was added to 100 mL of autoclaved seed medium, which included 20 g of glucose, 3 g of yeast extract, 10 g of peptone, 4 g of KH_2_PO_4_, 0.5 g KCl and 0.01 g FeSO_4_·7H_2_O. The inoculated seed medium was cultivated in an incubator shaker (HNY-211B, Tianjin, China) at 30 °C, 180 rpm for 28 h. The batch fermentation medium contained Chinese jujube pulps (5%, *w*/*v*), NaNO_3_ (0.2%, *w*/*v*), KH_2_PO_4_ (0.1%, *w*/*v*) and MgSO_4_·7H_2_O (0.1%, *w*/*v*). Notably, Chinese jujube first had the kernel removed and was homogenized with a knife mill (GRINDOMIX GM 200, RETSCH, Haan, Germany). Subsequently, a total of 10 mL seed culture was transformed into 1 L steam-sterilized fermentation medium and inoculated at 28 °C, 220 rpm for 9 days.

### 2.3. Extraction and Analysis of Phenolic Components

In total, 20 mL of fermented samples was extracted with 20 mL of 80% methanol at 40 °C for 30 min by ultrasonic extraction (SK2510LHC, 250 W, Shanghai Kedao Ultrasonic Instrument Co., Ltd. (Shanghai, China)). Then, the suspension was filtered with 0.22 μm syringe filter and freeze-dried to solid form for TSPC and TSFC determination. The residues were further digested and extracted for ISPC analysis by the previous reported methods [10]. Finally, both of the sample extracts were stored at −25 °C before analysis. The contents of ISPC and TSPC were determined by Folin-Ciocalteu method [24] and were expressed as gallic acid as equivalents in milligrams per gram (mg GAE/g). TSFC contents were measured by the NaNO_2_-AlCl_3_ method [19] and were expressed as rutin as equivalents in milligrams per gram (mg RE/g).

### 2.4. Qualitative and Quantitative Analysis of Phenolic Composition

The phenolic constituents of the fermented Chinese jujube were determined by the Liquid chromatograph-Mass spectrometer (LC-MS) method, as reported by our group [25]. Briefly, each part of the polyphenol extract was analyzed using HPLC (Shimadzu Corporation, Kyoto, Japan) coupled to a QTRAP5500 triple quadrupole linear ion trap mass spectrometer (AB Sciex, Foster City, CA, USA). Intertsil OSD-4C_18_ (150 mm × 3.0 mm, 3.5 μm) column was used to perform the chromatographic separation of 5 μL of each sample injected into a gradient system at a flow rate of 0.7 mL/min. The mobile phase consisted of 0.1% formic acid in deionized water (A) and methanol (B). Samples were eluted according to a linear gradient: 0–1 min, 25% solvent B; 1–6.5 min, 25–95% solvent B; 6.5–13 min, 95–25% solvent B. The column was operated at 40 °C throughout the total runtime.

The mass spectrometer was operated in positive or negative ion mode at 600 °C and the conditions of MS analysis were as follows: curtain gas (CUR) 35 psi, nebulizer gas (GS1) 60 psi, auxiliary gas (GS2) 65 psi, ionization voltage 4.5 kV. The phenolic material was fragmented and scanned using a multiple reaction monitoring (MRM) scanning method. Qualitative analysis of polyphenols was performed by the local database constructed by the Analyses and Testing Center of Northwest A&F University. Quantification was achieved by injection of solutions of the corresponding standard (i.e., quercetin, *p*-coumaric acid, ferulic acid, kaempferol, catechins, chlorogenic acid, rutin, protocatechuic acid, *p*-hydroxybenzoic acid, quercetin-3-*O*-glucuronide, hyperoside, luteolin).

### 2.5. RNA Extraction, Data Processing and Analysis

First, 5 mL liquid fermentation broth on the 2nd (Phase2) and 7th (Phase7) days was centrifuged at 12,000 rpm for 5 min at 4 °C, and the pellet was collected and washed with sterilized water twice. Then, RNA extraction, quantification and transcriptome sequencing were all performed at Novogene Corporation (Tianjin, China). After filtering out adapters and low-quality sequences, the clean reads were mapped against the seven public databases (NR: NCBI non-redundant protein sequences; Nt: NCBI nucleotide sequences; Swiss-Prot: Swiss protein database; KEGG: Kyoto Encyclopedia of Genes and Genomes; COG/KOG: COG: Clusters of Orthologous Groups of proteins, KOG: euKaryotic Ortholog Groups; GO: Gene Ontology; and Pfam: Protein family). The identification of differentially expressed genes (DEGs) was performed using the |log2-fold change| ≥ 1 and a corrected *p* ≤ 0.05 as thresholds.

The functional annotation of unigenes encoded as carbohydrate-active enzyme (CAZymes) was carried out by the carbohydrate-active enzymes (CAZymes) database (http://www.cazy.org/, accessed on 10 June 2021). To find CAZymes genes, the protein sequence extracted was used to search dbCAN (http://csbl.bmb.uga.edu/dbCAN/, accessed on 10 June 2021) database. Owing to the fact that more than one hit for each unigene was usually observed, the annotation of CAZymes for each unigene was performed with the following parameters [23]: e-value ≤ 1 × 10^−5^, the percentage of identical matches (pident) > 40% and the high-scoring segment pairs > 60 bits. Finally, the reference accession number of the CAZymes and family deposited in the CAZymes database were obtained for each identified CAZyme.

### 2.6. Determination of Carbohydrate-Hydrolyzing Enzymes Activities

Crude enzymes from the fermented Chinese jujube were extracted with deionized water (1:2 (*v*/*v*)), and subsequently centrifuged at 4 °C and 8000× *g* for 5 min. The supernatant was used for the enzyme activities analysis of *β*-glucosidase, xylanase and total cellulase. All of the above three hydrolyzing enzymes activities were determined spectrophotometrically (UV-1206 spectrophotometer, Shimadzu, Kyoto, Japan) using a method described by Bei et al. (2018) [9]. The results of each enzyme activity were all expressed as U/g.

### 2.7. Statistical Analysis

Three biological replicates were performed for each experiment. The principle components analysis (PCA) was performed by SIMCA-P package (Ver11.5; Umetrics Corp., Umea, Sweden). The heat map of hierarchical clustering analysis (HCA) was produced with MultiExperiment Viewer software (MeV, v4.8.1, Boston, MA, USA). SPSS 20 (Ver20.0 IBM Corp., Armonk, NY, USA) was applied for one-way analysis of variance (ANOVA) and correlation analysis of data. Significant difference between the samples was determined by Duncan’s test. Difference at *p* < 0.05 was considered to be significant.

## 3. Results and Analysis

### 3.1. Selection of Microorganism and Optimization of LSF Condition

**Figure 1:**
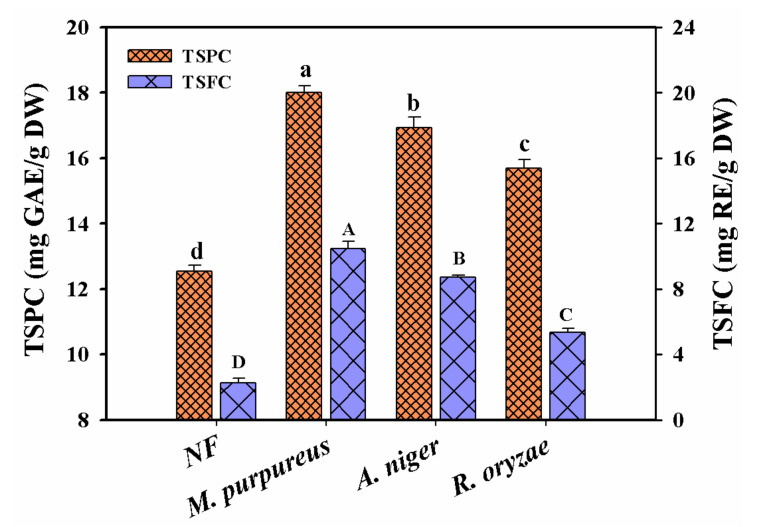
The contents of TSPC and TSFC in the fermented Chinese jujube by *R. oryzae*, *A. niger* and *M. purpureus*, respectively. Different superscripts (abcd/ABCD) indicated significant differences between samples at the different strains (*p* < 0.05). NF represented the unfermented samples.

In order to screen the suitable strains, three kinds of fungi strains (*R. oryzae*, *A. niger* and *M. purpureus*) were selected to evaluate their effects on the TSPC and TSFC contents in Chinese jujube fermentation, respectively. As seen in Figure 1, the contents of TSPC and TSFC showed a similar tendency in all selected strains, both of which reached the maximum values at the 7th day of fermentation and then decreased. The highest yields of TSPC and TSFC increased with 39.8% and 359.2% for *M. purpureus*, 31.4% and 282.1% for *A. niger*, 21.8% and 135.1% for *R. oryzae* from the initial values of 12.88 mg GAE/g DW and 2.28 mg RE/g DW, respectively. Therefore, *M. purpureus* was finally selected as the suitable strain in Chinese jujube fermentation.

**Figure 2:**
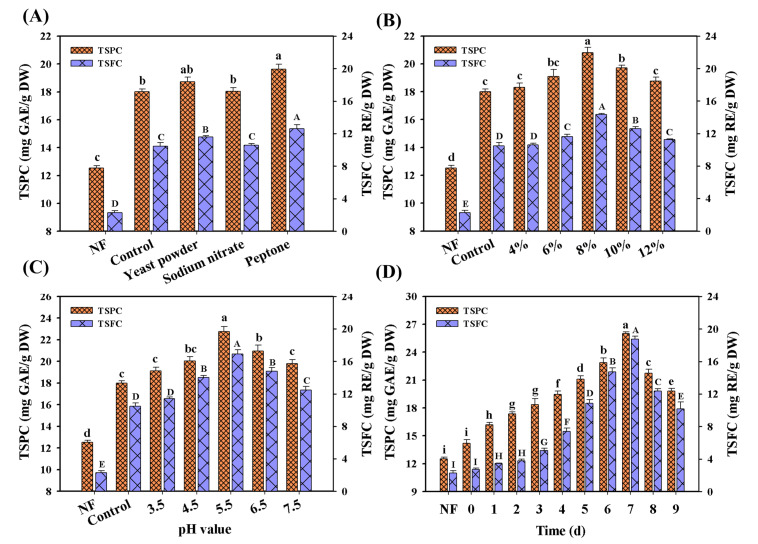
The optimization of fermentation parameters in LSF. (**A**) Nitrogen source, (**B**) inoculation level, (**C**) the initial pH value, (**D**) the combined optimal conditions. NF represents non-fermented Chinese jujube sample. Different superscripts (abcdefghi/ABCDEFGHI) indicated significant differences between samples at the different fermented conditions (*p* < 0.05). NF represented the unfermented samples.

As the previous description puts forth, the fermentation conditions played important roles in the bioconversion or liberation of polyphenol compounds in several food materials (e.g., kiwifruit, oats, berry pomaces) [22,26,27]. Thus, the fermentation parameters of *M. purpureus* were first optimized during LSF (Figure 2), including the initial pH value, inoculation level and nitrogen sources. Among all the selected nitrogen sources, the obvious improvement of TSPC and TSFC contents can be observed in all the fermented Chinese jujube. After feeding peptone, therefore, the highest contents of TSPC and TSFC could reach 19.64 mg GAE/g DW and 12.62 mg RE/g DW (Figure 2A), 52.5% and 453.5% of enhancement compared to that of the unfermented sample, respectively. This was consistent with the previous reports that peptone was the suitable nitrogen source for the strain growth and the bioactive compounds production of *Monascus* [28]. Meanwhile, the increase of TSPC and TSFC contents in all feeding nitrogen medium further confirmed that the low nitrogen content of a medium was a barrier in the release of free phenolic compounds during LSF/SSF [27]. In addition to the optimization of nitrogen sources, the optimized pH value and inoculation level were 5.5 and 8% (*v*/*v*) (Figure 2B,C), with 22.76 mg GAE/g DW and 20.82 mg GAE/g DW for TSPC, 16.88 mg RE/g DW and 14.33 mg RE/g DW for TSFC, respectively. Under the above optimal conditions, the final yields of TSPC and TSFC increased by 102.1% (26.02 mg GAE/g DW) and 722.8% (18.76 mg RE/g DW) at the 7th day compared to that of the non-fermented sample, respectively (Figure 2D). Notably, the significant decrease of TSPC and TSFC contents was found after 7 days fermentation. In the previous studies, the similar phenomenon had also been reported [13,19]. A possible reason is that most of the phenolic compounds had been degraded in the later phase of *M. purpureus* fermentation. For example, it has been confirmed that quercetin and other phenolic compounds can be obviously degraded at the later phase of *A. niger* fermentation in tartary buckwheat leaves [13]. At the later phase of fermentation, in fact, the depletion of nutrients (e.g., carbon and nitrogen sources) would severely restrict the strain growth. To obtain the enriched nutrients, the potential carbon sources (e.g., polyphenols) might be used to maintain their normal cell life activities by a series of enzymatic reactions. In this work, notably, over 90% of the total sugars had been consumed after 7 days fermentation. These results also confirmed the above-mentioned speculation, which would be verified in the further work.

### 3.2. Qualitative Analysis of Phenolic Composition during LSF

**Figure 3:**
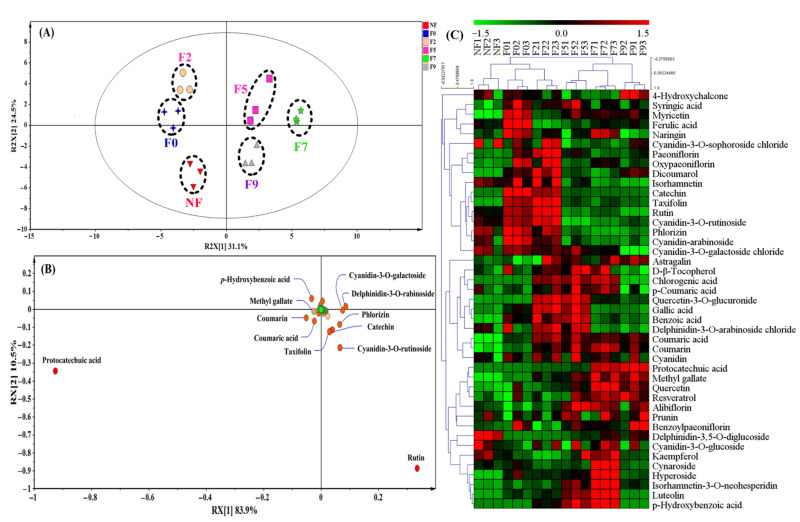
Qualitative and quantitative analysis of polyphenol compounds in fermented Chinese jujube by *M. purpureus*. (**A**): PCA score plot, (**B**): PCA loading plot, (**C**): heat map of HCA analysis. F0, F2, F5, F7 and F9 represented the samples collected at 0, 2, 5, 7 and 9th day of the fermentation, respectively. NF represented the unfermented samples.

A total of 43 polyphenol compounds were identified by LC-MS (Figure 3) in the fermented Chinese jujube. According to the PCA plot (Figure 3A), the obvious separation between the fermented and unfermented samples indicated that the obvious changes of polyphenol compounds profiling had taken place in the processing of fermentation. As seen in Figure 3B, 12 compounds were identified successfully, which can be recognized as the potential key biomarkers for the fermented Chinese jujube (Figure 3B). It is worth noting that these compounds could be grouped into two categories based on their changes of the relative abundances. One category is that of the phenolic compounds located in the right of the *Y*-axis (i.e., catechin, taxifolin, rutin, cyanidin-3-*O*-rutinoside, phlorizin, cyanidin-3-*O*-galactoside and delphinidin-3-*O*-arabinoside). For these compounds, an obvious feature is that the enhancement of its relative abundance was mainly derived from autoclaving (Figure 3C). In fact, the influence of the high pressure and heating treatment on the release of polyphenol had been investigated before. For instance, the content of catechin could increase over 640% for the roasted beans at 120 °C than for that of the unroasted cacao beans [27]. These results suggest that these above-mentioned phenolic compounds within Chinese jujube were sensitive to high temperature or pressures in the sterilization process, which was also consistent with the previous research [29].

The other category is that of the phenolic compounds that are located at the left of the *Y*-axis (Figure 3B), which displayed the gradually elevating tendencies during *M. purpureus* fermentation (Figure 3C), including *p*-hydroxybenzoic acid (21.34-fold, 7d), protocatechuic acid (11.41-fold, 7d), coumaric acid (11.06-fold, 7d), coumarin (2.64-fold, 7d) and methyl gallate (2.29-fold, 7d). Interestingly, most of these phenolic compounds were hydroxycinnamic acid derivatives. Thus, *p*-hydroxybenzoic acid and protocatechuic acid displayed the highest relative abundance at the 7th days of fermented Chinese jujube. In fact, the changes of these phenolic compositions and contents were mainly attributed to the action of the carbohydrate-hydrolyzing enzymes produced by microorganisms. For example, catechin and gallic acid could be metabolized to protocatechuic acid by the action of catechin oxygenase or tannase [22,30]. The derivatives of hydroxybenzoic acid (C_6_-C_1_) could be produced by cleavage of C_2_ fragment from phenylpropanoids (e.g., coumarin) [31]. Additionally, it was also found that *p*-coumaric acid derived from conjugated phenolic acids can be released by feruloyl esterase (also named ferulic acid esterase), and further formed protocatechuic acid [22]. Therefore, the identification of these hydroxycinnamic acid derivatives might also imply that these compounds might be the key metabolic nodes in the degradation and conversion of polyphenol compounds for the fermented Chinese jujube.

### 3.3. Quantitative Analysis of Individual Phenolic Compounds

To further confirm the reliability of the above qualitative analysis, the quantitative determination of individual phenolic compounds (IPCs) was carried out during Chinese jujube fermentation (Table 1). Among all the detected IPCs, rutin displayed the highest content (28.94 μg/g) in the unfermented samples. This was in accordance with the previous reports that rutin was one of the main phenolic compounds in Chinese jujube [1]. After autoclaving, notably, the highest contents of rutin could reach 59.8 μg/g and then decreased along with fermentation. With rutin, the lower contents of protocatechuic acid and chlorogenic acid were observed in unfermented samples, both of which increased continuously to 109.2 μg/g and 44.71 μg/g at the 7th day of fermentation, respectively. As the highest abundant phenolic compound in the fermented Chinese jujube, it was reported that protocatechuic acid could not only be synthesized by the biotransformation of catechin (or gallic acid), but could also be formed from the degradation of anthocyanin [14]. Notably, the relative abundances of most identified anthocyanin (especially for cyanidin derivatives, e.g., cyanidin-3-*O*-rutinoside and cyanidin-3-*O*-galactoside) showed the down-tendencies with the prolonging of the fermentation (Figure 3C). In addition to the degradation of catechin (from 5.72 μg/g to 0.40 μg/g), therefore, the transformation of anthocyanin within Chinese jujube peels might also be a potential source in the enhancement of the protocatechuic acid level during fermentation. As the phenolic compound with the highest change multiplied, the contents of *p*-hydroxybenzoic acid increased to 19.53 μg/g at the 7th day from 1.39 μg/g of the unfermented sample. The higher contents of these hydroxycinnamic acid derivatives further confirmed that they might be the key metabolic intermediates in the bioconversion or degradation of polyphenol compounds during Chinese jujube fermentation.

### 3.4. Transcriptome Sequencing and Carbohydrate-Active Enzymes Identification

**Figure 4:**
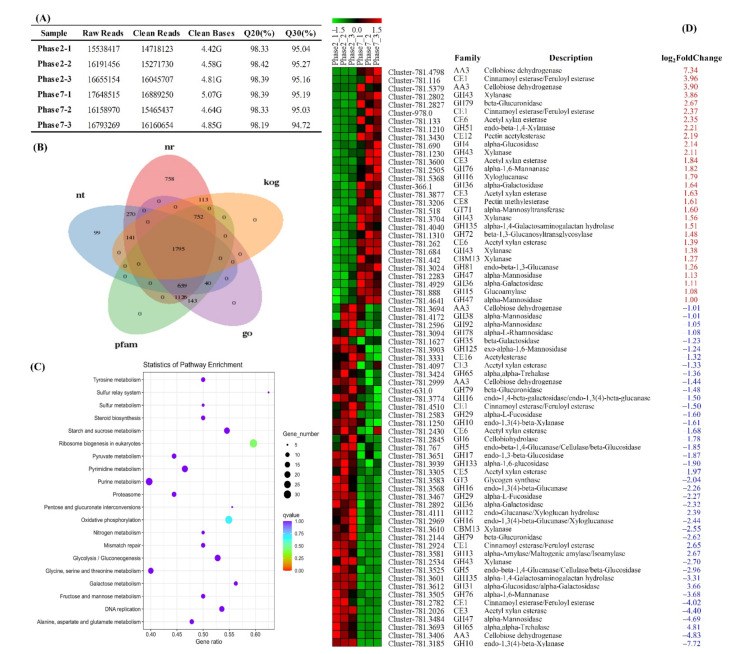
Functional annotation and classification of unigenes in transcriptomics analysis. (**A**) Summary of transcriptome sequencing data quality, (**B**) Venn diagram illustrating shared and unique unigenes annotated in public database, (**C**) KEGG enrichment analysis of all the identified DEGs and (**D**) CAZymes annotation of DEGs. The red and blue numbers in Figure 4D represented the up-regulated and down-regulated multiples of these identified CAZymes, respectively.

To better understand the action mechanism of *M. purpureus* on phenolic compounds metabolism, RNA sequencing of transcriptomes was performed in the fermented Chinese jujube samples at 2nd day (Phase2) and 7th day (Phase7), respectively. A total of 28.37 G clean data were generated from all the detected samples after rigorous quality assessment and data filtering (Figure 4A). Meanwhile, all of the collected unigenes were compared against the seven public databases (Figure 4B, NR, Nt, Swiss-Prot, KEGG, COG/KOG, GO and Pfam), and 6641 unigenes were annotated finally. Using the |log2-fold change| ≥ 1.0 and *p*-value ≤ 0.05 as the thresholds, 1945 DEGs were identified with more than 1059 upregulated and more than 886 downregulated unigenes. According to the results of KEGG pathway enrichment for DEGs (Figure 4C), several metabolic pathways within carbohydrate metabolism were identified, such as starch and sucrose metabolism, glycolysis/gluconeogenesis, galactose metabolism, fructose and mannose metabolism, pyruvate metabolism and pentose and glucuronate interconversions. The enhancement of the above-mentioned carbohydrate metabolic pathways could not only ensure the growth and reproduction of strains, but also provide suitable conditions for the synthesis and secretion of CAZymes during *M. purpureus* fermentation.

CAZymes are involved in the assembly and breakdown of complex carbohydrates, involving oligosaccharides or polysaccharides as well as glycoconjugates to nucleic acids, proteins, lipids, polyphenols and other natural compounds [32]. Among all the identified DEGs, a total of 71 genes are predicted to encode CAZymes (Figure 4D), involving 46 families of glycoside hydrolases (GHs), 16 families of carbohydrate esterases (CEs), 5 families of auxiliary activities (AAs), 2 families of carbohydrate-binding modules (CBMs) and 2 families of glycosyltransferases (GTs). Interestingly, GHs were found to be the most abundant category, which were involved in hemicellulose degradation (e.g., GH35, GH36, GH43), cellulose branching/debranching (e.g., GH5, GH16, GH31), starch branching/debranching (e.g., GH4, GH13), polysaccharides degradation (e.g., GH38, GH51, GH92) and so on. In the previous literature, it has been reported that phenolic compounds are usually conjugated with sugars (i.e., phenolic glycosides) [14], which could be degraded by the above-mentioned glycoside hydrolases (GHs), such as xylanase, beta-galactosidase, alpha-galactosidase, alpha-glucosidase, alpha-L-rhamnosidase and so on. During the fermentation of Chinese jujube, some phenolic glycosides (e.g., alibiflorin, astragalin, quercetin-3-*O*-glucuronide, delphinidin-3,5-O-diglucoside, cyanidin-3-*O*-glucoside, isorhamnetin-3-*O*-neohesperidin, hyperoside and cynaroside) were identified and showed higher relative abundance at the beginning of fermentation, and decreased obviously with the prolonging of fermentation (Figure 3C). Therefore, the abundant expression of GHs might be responsible for the transformation and degradation of these phenolic glycosides in fermented Chinese jujube.

In addition to GHs family, 16 families of CEs, catalyzing the O- or N-deacylation of substituted saccharides, were also identified and assigned to the families CE1, CE3, CE5, CE6, CE8, CE12 and CE16. Thus, the most numerous families were CE1 (5 proteins) and CE3 (4 proteins). According to the annotation from the CAZy database, CE1 encoding cinnamoyl esterase/feruloyl esterase could catalyze the hydrolysis of the 4-hydroxy-3-methoxycinnamoyl (feruloyl) group from an esterified sugar. In fact, the previous research had confirmed that acetyl xylan esterase (CE4) played an important role in promoting the hydrolysis of cellulose [33]. Notably, hydroxycinnamic acid derivatives (e.g., ferulic, *p*-coumaric, coumaric and caffeic acids) are the important constituents of plant cell walls [14], which can be released under the action of these above enzymes secreted by *M. purpureus*. Therefore, the enhancement of *p*-coumaric acid, coumaric acid and ferulic acid at the beginning of fermentation could be derived from the release (or degradation) of ISPC bounded in plant cell walls.

In the previous reports, CAZymes within the families or subfamilies of AAs also played important roles in the structure conversion of phenolic compounds bearing side chains at the aromatic ring, such as AA1 (laccase), AA4 (vanillyl-alcohol oxidase), AA6 (benzoquinone reductase) and so on [18]. For example, the polymerization of a wide range of phenolic substrates (e.g., catechin, catechol, gallic acid, ferulic acid and rutin) was catalyzed by laccase. Vanillyl-alcohol oxidase showed higher activities in the oxidation of a wide range of para-substituted phenolic compounds. The reduction of quinones to hydroquinones could be catalyzed by benzoquinone reductase during the biodegradation of aromatic compounds. Unfortunately, only AA3 (cellobiose dehydrogenase) was identified in all the DEGs, which suggested that the hydrolysis of cellulose might have happened significantly. By the further function annotation of the rest of the unigenes, however, some genes that encoded the above-mentioned proteins were also found, all of which displayed high-expression abundance in both of the two selected periods, including three laccases (Cluster-781.3951, Cluster-781.4137 and Cluster-781.5437), one benzoquinone reductase (AA6, Cluster-781.2387) and four vanillyl-alcohol oxidases (Cluster-781.3841, Cluster-781.2927, Cluster-728.1 and Cluster-728.0). Therefore, the synthesis and secretion of these enzymes by *M. purpureus* resulted in the changes of polyphenol and benzenoids during Chinese jujube fermentation.

### 3.5. Dynamic Changes of ISPC Contents and Enzymes Activities Verification

**Figure 5:**
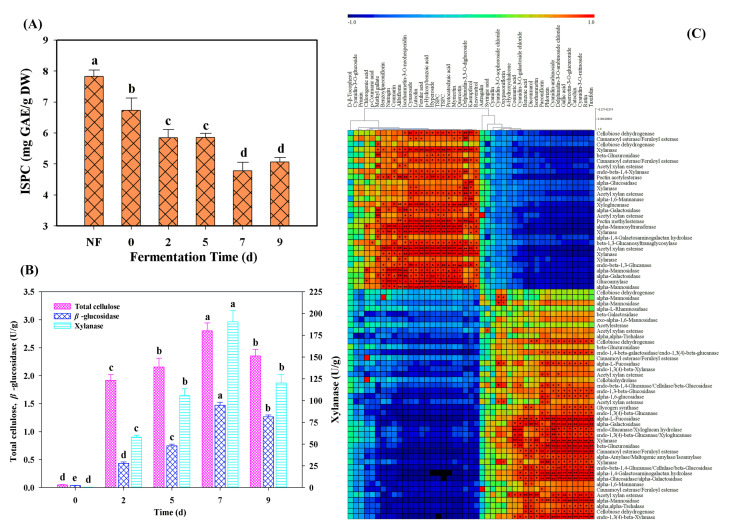
The contents of ISPC content (**A**), carbohydrate-hydrolyzing enzymes activities (**B**) and the correlation analysis among individual phenolic compounds, TSPC, TSFC and carbohydrate-hydrolyzing enzymes (**C**). NF stands for non-fermented Chinese jujube. ** and * represents the significant level at *p* < 0.01 and *p* < 0.05, respectively. Different superscripts (abcde) indicated significant differences between samples at the different fermented times (*p* < 0.05).

To further confirm the potential bioconversion of polyphenol compounds, the ISPC contents and the activities of part carbohydrate-hydrolyzing enzymes were measured during *M. purpureus* fermentation. As shown in Figure 5A, the obvious decrease of ISPC contents (from 6.73 to 4.78 mg GAE/g DW) was observed from 0 to the 7th day of fermentation. The degradation of ISPC could provide the potential sources for the enhancement of the free and soluble conjugated phenolic compounds. Among all the selected carbohydrate-hydrolyzing enzymes, the highest enzyme activities were all found at the 7th day of fermentation, and then decreased. It is worth noting that xylanase showed the highest activities (190.55 U/g) compared to that of cellulase (2.80 U/g) and *β*-glucosidase (1.47 U/g) (Figure 5B). Interestingly, several genes (e.g., Cluster-781.2534, Cluster-781.3610, Cluster-781.3704, Cluster-781.2802) encoding xylanase had also showed stronger correlation with the most identified phenolic compounds in this work (Figure 5C). Meanwhile, the other enzymes (e.g., glucuronidase, galactosidase, mannosidase) had also displayed significant correlation with the most determined phenolic compounds. These results further confirmed that the phenolic compounds conjugated with xylan (or cellulose/lignin) and other sugars (e.g., glucose, galactose and rhamnose) were degraded during *M. purpureus* fermentation.

## 4. Discussion

**Figure 6:**
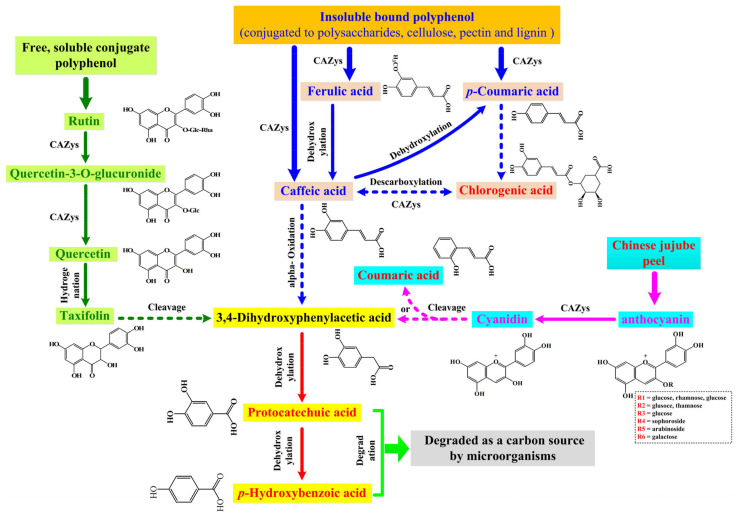
Proposed catabolic pathways of polyphenols degradation and transformation in the fermented Chinese jujube by *M. purpureus*. The solid and dotted arrows represented the one-step and multi-step reactions, respectively.

As an attractive biotransformation method of bioactive compounds (e.g., polyphenol, polypeptide, polysaccharose), microbial fermentation has been widely applied in different food materials to improve the bioactive functions of the final products. Therefore, the high efficiency of fermentation on the release of polyphenol compounds has been described in some plant materials, such as kiwifruit [22], black rice [14], oats [26], and so on. However, the potential biotransformation mechanism of polyphenol compounds under microbial fermentation has not been clarified comprehensively until now. For example, the obvious increase of some hydroxycinnamic acid derivatives (e.g., chlorogenic acid, protocatechuic acid and *p*-hydroxybenzoic acid) contents after microbial fermentation had been found in several previous reports, but the potential conversion pathways of these compounds were only described partially [22,34,35]. To solve this problem, in this work, the method of combining transcriptomics and phenolic compounds profiles was used to clarify the potential mechanism of the biotransformation in polyphenol compounds in the fermented Chinese jujube by *M. purpureus* (Figure 6). The details are as follows:

(1) The degradation of insoluble bound phenolic acids was the main source in the augmentation of chlorogenic acid, protocatechuic acid and *p*-hydroxybenzoic acid level. According to the previous description, ferulic, *p*-coumaric and caffeic acids are typical constituents of plant cell walls, which are conjugated with cellulose, lignin and proteins by ester linkages [14]. The corresponding ester bonds could be broken by CE1 (i.e., cinnamoyl esterase/feruloyl esterase) produced by *M. purpureus* (Figure 4D), and could transform into free soluble phenolic acids (e.g., caffeic acids). Subsequently, caffeic acids can be transformed into *p*-coumaric acid by dehydroxylation, and then could form protocatechuic acid by a series of alpha-oxidation and dehydrogenation steps (Figure 6). The specific mechanism of transformation in the above-mentioned pathways has been confirmed in *Pycnoporus cinnabarinus* MUCL39533 [35] and *Bacillus megaterium* [36], respectively.

(2) The degradation of rutin might be another crucial source for the enhancement of protocatechuic acid and *p*-hydroxybenzoic acid level. As one of the major phenolic compounds in Chinese jujube, the obvious decrease of rutin contents has been observed in fermented Chinese jujube (Table 1). Rutin, as a type of flavonoid glycoside, could be degraded to quercetin-3-*O*-glucuronide (or quercetin) by GHs (e.g., glucosidase, rhamnosidase). That is why the obvious enhancement of quercetin and taxifolin contents was found in the processing of fermentation (Figure 3C and Table 1). Further, the enol carbon C-3 of quercetin can be transformed to the carboxyl group of 3,4-dihydroxyphenylacetic acid, and could form protocatechuic acid (or *p*-hydroxybenzoic acid). The above-mentioned degradation of quercetin within the above pathway has also been reported in *Eubacterium ramulus* [37].

(3) The last supposed pathway is anthocyanin degradation. In Chinese jujube fermentation, several kinds of anthocyanin were identified, and most of them were cyanidin glycoside derivatives (Figure 3C, e.g., cyanidin-3-*O*-glucoside, cyanidin-3-*O*-rutinoside and cyanidin-3-*O*-galactoside). Similar to rutin, a part of glycoside could be hydrolyzed to cyanidin by GHs produced by *M. purpureus* (Figure 4D). Then, cyanidin can be decomposed by two pathways to end up with either coumaric acid or benzoic acid derivatives (e.g., protocatechuic acid), respectively [38]. However, while it has been reported that anthocyanin was easy to be degraded under thermal treatments [37], some kinds of anthocyanin were still identified in fermented Chinese jujube (Figure 3C). So, the degradation of the undecomposed anthocyanin might also be another potential source for the increase of protocatechuic acid level in this work. Meanwhile, it might be the potential cause of the higher enhancement of coumaric acid level (11.06-fold, Figure 3C) during the processing of Chinese jujube fermentation.

In conclusion, the conversion and degradation of polyphenol compounds in fermented Chinese jujube by *M. purpureus* are a very complex process. Only parts of hydroxycinnamic acid derivatives (i.e., chlorogenic acid, protocatechuic acid and *p*-hydroxybenzoic acid) with higher contents under *M. purpureus* fermentation were analyzed comprehensively in this work. The detailed process of the rest of the polyphenol compounds’ degradation (or conversion) has not been discussed, and it will also be the focus of further work. The findings of this work could not only be of benefit for the development of Chinese jujube functional products, but could also advance knowledge of the functions of *M. purpureus* in the metabolism of polyphenol compounds.

## Figures and Tables

**Table 1:** Contents of the identified phenolic compounds in fermented Chinese jujube by *M. purpureus*.

Fermentation Time (d)	NF	0d	2d	5d	7d	9d
**Catechins**	0.26 ± 0.07 cd	5.72 ± 0.39 a	4.22 ± 0.10 b	0.54 ± 0.05 c	0.40 ± 0.05 cd	0.07 ± 0.04 d
**Ferulic acid**	1.02 ± 0.59 b	1.47 ± 0.06 a	0.22 ± 0.04 b	0.55 ± 0.06 b	0.72 ± 0.16 b	0.68 ± 0.40 b
**Chlorogenic acid**	7.41 ± 0.68 b	8.05 ± 0.35 b	41.40 ± 0.63 a	42.60 ± 1.92 a	44.71 ± 4.51 a	2.10 ± 0.20 c
**Rutin**	28.94 ± 0.44 c	50.60 ± 0.45 b	59.80 ± 0.40 a	18.26 ± 0.07 f	11.15 ± 0.06 e	5.52 ± 0.18 d
***p*-Coumaric acid**	2.58 ± 0.25 d	2.97 ± 0.19 d	4.70 ± 0.09 c	5.28 ± 0.42 b	6.57 ± 0.09 a	4.66 ± 0.12 c
**Quercetin**	0.59 ± 0.05 b	0.49 ± 0.12 b	1.35 ± 0.78 ab	1.50 ± 0.04 ab	1.85 ± 0.58 a	0.52 ± 0.10 b
**Protocatechuic acid**	6.52 ± 0.12 e	9.83 ± 0.07 d	18.29 ± 0.65 c	26.39 ± 1.44 b	109.20 ± 0.42 a	3.43 ± 0.15 f
***p*-Hydroxybenzoic acid**	1.39 ± 0.04 d	1.03 ± 0.14 e	8.66 ± 0.03 c	15.12 ± 0.13 b	19.53 ± 0.17 a	0.53 ± 0.07 f
**Kaempferol**	2.05 ± 1.92 ab	0.24 ± 0.01 b	0.42 ± 0.31 b	1.55 ± 0.27 ab	2.85 ± 0.23 a	1.88 ± 1.05 ab
**Quercetin-3-*O*-glucuronide**	0.03 ± 0.01 d	0.04 ± 0.01 d	0.65 ± 0.05 a	0.68 ± 0.12 a	0.25 ± 0.01 b	0.15 ± 0.04 c
Hyperoside *	ND	0.11 ± 0.13 b	0.02 ± 0.01 b	0.10 ± 0.04 b	0.63 ± 0.03 a	ND
Luteolin *	ND	0.58 ± 0.22 c	0.74 ± 0.11 c	1.81 ± 0.21 b	2.85 ± 0.21 a	0.10 ± 0.01 d

* represents that the contents unit of these phenolic compounds is μg/100 g; the contents unit of the rest of the phenolic compounds is μg/g. Different lower case letters at the same column correspond to significant differences at *p* < 0.05. NF stands for non-fermented sample, ND represents not detected.

## Data Availability

The data presented in this study are available on request from the corresponding author.
